# Remarkable plasticity of Na^+^, K^+^-ATPase, Ca^2+^-ATPase and SERCA contributes to muscle disuse atrophy resistance in hibernating Daurian ground squirrels

**DOI:** 10.1038/s41598-017-10829-6

**Published:** 2017-09-05

**Authors:** Quanling Guo, Xin Mi, Xiaoyong Sun, Xiaoyu Li, Weiwei Fu, Shenhui Xu, Qi Wang, Yasir Arfat, Huiping Wang, Hui Chang, Yunfang Gao

**Affiliations:** 1 Key Laboratory of Resource Biology and Biotechnology in Western China (Northwest University), Ministry of Education, Xi’an, 710069 China; 2 Shaanxi Sci-Tech University, School of Biological Science and Engineering, Hanzhong, 723001 China

## Abstract

We investigated cytosolic calcium (Ca^2+^) and sarcoplasmic reticulum Ca^2+^ regulation in skeletal muscle fibers of hibernating Daurian ground squirrels (*Spermophilus dauricus*), non-hibernating hindlimb-unloaded (HLU) squirrels, and HLU rats to clarify the molecular mechanisms involved in preventing muscle atrophy in hibernators. The Na^+^, K^+^-ATPase and Ca^2+^-ATPase activities in the soleus muscle (SOL) of squirrels were maintained in hibernation, decreased during interbout arousal (IB-A), and increased to autumn/pre-hibernation (AUT/Pre-H) levels in torpor after interbout arousal (Post-IBA), whereas activities in the extensor digitorum longus muscle (EDL) were stable during hibernation, but increased during post-hibernation (Post-H). Activities increased in the SOL of HLU rats, but were stable in HLU squirrels. Sarco/endoplasmic reticulum Ca^2+^-ATPase (SERCA) activity in the SOL decreased in IB-A squirrels, but returned to AUT/Pre-H levels in the Post-IBA group; no significant changes were found in the EDL. SERCA activity increased in the EDL of HLU squirrels and SOL of HLU rats. Compared with AUT/Pre-H, SERCA type 2 protein expression increased in the SOL and EDL of IB-A and Post-IBA squirrels, but increased in the SOL only in HLU animals. We also describe the protein kinase A changes in this paper. Thus, hibernating ground squirrels displayed remarkable Na^+^, K^+^-ATPase, Ca^2+^-ATPase, and SERCA plasticity.

## Introduction

Following sustained unloading or disuse, mammalian skeletal muscles can show considerable loss or deficiency in contractile proteins. Previous studies have indicated that increased protein catabolism might be a key driver of this loss^1–4^. Proteolytic pathways mainly include the calpain pathway, cysteine-containing aspartate-specific proteases-3 (caspase-3) pathway, ubiquitin proteasome pathway, and autophagy^5^. The calpain family consists of calcium-dependent proteases, which play critical roles in the initial stage of protein structure degradation^6–8^. Calpains activated by calcium (Ca^2+^) can instigate myofibrillar protein degradation by supporting the release of molecules that can be further damaged by proteasomes^5^. Skeletal muscle disuse induces cytosolic Ca^2+^ overload, which can activate ubiquitin proteasome by activating calpains, thereby accelerating skeletal muscle protein degradation and muscle atrophy^9, 10^. Furthermore, the activities of Ca^2+^-ATPase, Na^+^ and K^+^-ATPase, as well as the expression levels of sarcoplasmic reticulum Ca^2+^-ATPase (SERCA), can be altered in the soleus (SOL) muscle following disuse^2, 11, 12^. Therefore, alterations and disruptions in cytosolic Ca^2+^ regulation and an imbalance in Ca^2+^ homeostasis could be crucial mechanisms of muscle disuse atrophy.

Daurian ground squirrels (*Spermophilus dauricus*) belong to the Sciuridae family. Interestingly, those within the Sciuridae family that hibernate display only minimal loss of skeletal muscle mass, as well as improved wet muscle to total body mass ratios and unmodified fiber cross-sections, during their winter quiescence^13–15^. Studies on the underlying mechanism related to this have been conducted in certain hibernating species. For example, the down-regulation of transcription factor FOXO1 and MAFbx mRNA in *Spermophilus lateralis*, another ground squirrel species, might play a role in reduced muscle deterioration^16^. The expression of MuRF1, which is stable in the SOL muscle of Daurian ground squirrels during hibernation, might help prevent atrophy by regulating the ubiquitination of muscle proteins^13^. Furthermore, muscle properties responsible for atrophy resistance in species such as torpid bats (*Murina leucogaster ognevi*) might be related to constant proteolysis in combination with oscillatory anabolic activity (e.g., p-mTOR) and the stability or up-regulation of molecular chaperones such as heat shock proteins^17, 18^. In our previous research, Daurian ground squirrels did not demonstrate significant cytosolic Ca^2+^ overload induced by disuse during hibernation inactivity, as has been observed in non-hibernating animals, and cytosolic Ca^2+^ homeostasis was maintained in their slow-twitch SOL and fast-twitch EDL muscles^19^. The remarkable preservation of Ca^2+^ homeostasis in skeletal muscles of hibernators could prevent the activation of calpains, which accelerate protein degradation and skeletal muscle atrophy^9, 10^. Thus, the maintenance of cytosolic Ca^2+^ homeostasis might be a critical mechanism of resistance to disuse atrophy in hibernators. However, the mechanisms of Ca^2+^ regulation have not yet been clarified in hibernators.

Calcium homeostasis is important to maintain the intracellular environment in mammals, and within skeletal muscle fibers is the basis for maintaining normal physiological function. Calcium related channels and ion pumps in mitochondria, sarcoplasmic reticula, and plasma membranes play important roles in the maintenance of cytosolic Ca^2+^ homeostasis. Previous studies have demonstrated that cytosolic Ca^2+^ overload is associated with mitochondrial-mediated apoptosis in non-hibernators^20^. In contrast, no apoptosis was observed in skeletal muscle fibers during hibernation in our recent study^19^, suggesting that mitochondrial Ca^2+^ levels might be maintained during hibernation and mitochondria might not be used as a Ca^2+^ pool to buffer possible cytosolic Ca^2+^ overload in the skeletal muscle of hibernators.

The ryanodine receptor (RyR) and sarco/endoplasmic reticulum calcium ATPase (SERCA) pump are Ca^2+^ regulated proteins in the endoplasmic reticulum (ER) and the more specialized sarcoplasmic reticulum (SR) in muscle cells. The dynamic release of Ca^2+^ from these organelles is mediated by the RYR.

Ca^2+^-ATPase reduces the Ca^2+^ concentration in the cytoplasm and is located in the cell membrane where Ca^2+^ is pumped out of the cell consuming ATP and in the sarcoplasmic reticulum, into which Ca^2+^ is pumped. It has been reported that 92% of recovery of cytosolic Ca^2+^ concentration in the diastolic phase depends on the uptake of Ca^2+^ via SERCA2a^21^.

Here, RYR, which can result in increased cytosolic Ca^2+^, together with no Ca^2+^ overload in skeletal muscle fibers after hibernation, which might not be a critical factor in the maintenance of cytosolic Ca^2+^, and SERCA, which plays a crucial role in the prevention of cytosolic Ca^2+^ overload, were investigated in the present study.

Na^+^, K^+^-ATPase is essential for establishing normal intracellular [Na^+^] and [K^+^] as well as transmembrane gradients crucial for many cellular functions, including cytosolic Ca^2+^ concentration regulation. Intracellular Na^+^ concentration is an important regulator of Ca^2+^ homeostasis through its influence on the Na^+^-Ca^2+^ exchanger (NCX). Na^+^, K^+^-ATPase can modulate NCX-mediated Ca^2+^ transport by establishing a shared microdomain of Na^+^ where [Na^+^]i can differ from that in bulk cytosol^22^. NCX participates in the Ca^2+^ outflow across the plasma membrane, which is an important factor in maintaining cytosolic Ca^2+^ at normal physiological levels.

Thus, we hypothesized that hibernators would be able to maintain intracellular Ca^2+^ concentration in hibernation through regulating Na^+^, K^+^-ATPase, Ca^2+^-ATPase, and SERCA, among which SERCA might play the most important part in reducing the intracellular Ca^2+^ concentration. In other words, up-regulation of the major factors that reduce the cytosolic Ca^2+^ concentration might be the key mechanism in hibernating ground squirrels for maintaining cytosolic Ca^2+^ homeostasis and thus preventing disuse atrophy. Therefore, we investigated the regulatory proteins of cytosolic Ca^2+^ and SR Ca^2+^ in the skeletal muscle fibers of hibernating ground squirrels and hindlimb-unloaded ground squirrels and rats. We also assessed protein kinase A (PKA) to explore the regulating mechanism of SERCA activity, which is widely dependent on the phosphorylation of phospholamban (PLB) by PKA^23^.

## Results

### Effects of hibernation on Na^+^, K^+^-ATPase and Ca^2+^-ATPase activity in the SOL muscle of Daurian ground squirrels

**Figure 1:**
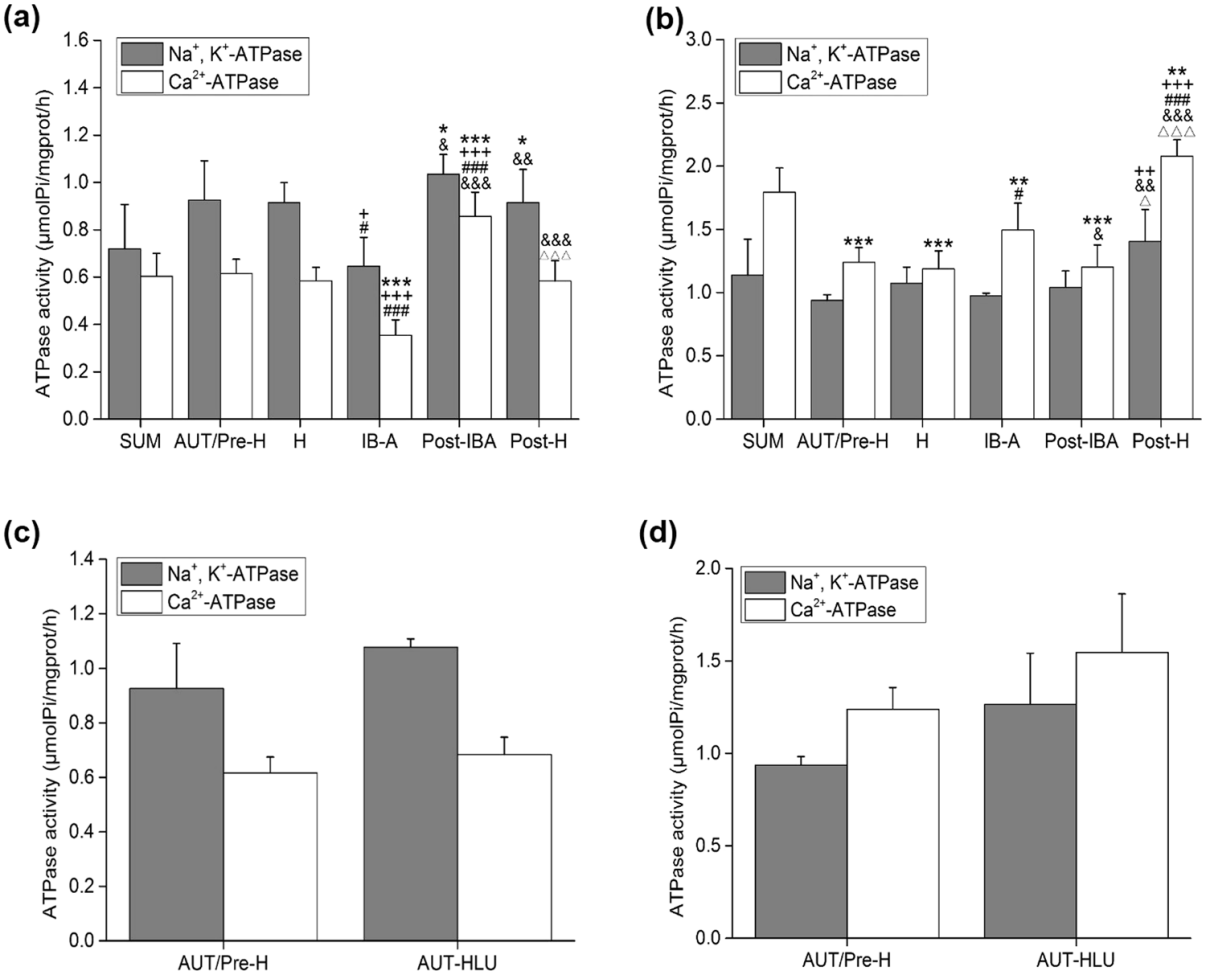
Effects of hibernation and hindlimb-unloading on Na^+^, K^+^-ATPase and Ca^2+^-ATPase activity in the SOL and EDL muscles of Daurian ground squirrels (mean ± SD). (**a**) Activities in the SOL muscle of hibernation squirrels. (**b**) Activities in the EDL muscle of hibernation squirrels. (**c**) Activities in the SOL muscle of hindlimb-unloaded squirrels. (**d**) Activities in the EDL muscle hindlimb-unloaded squirrels. *P < 0.05, **P < 0.01, ***P < 0.001, Compared with the SUM group; ^+^ *P* < 0.05, ^++^ *P* < 0.01, ^+++^ *P* < 0.001, Compared with the AUT/Pre-H group; ^#^ *P* < 0.05, ^###^ *P* < 0.001, Compared with the H group; ^&^ *P* < 0.05, ^&&^ *P* < 0.01, ^&&&^ *P* < 0.001, Compared with the IB-A group; ^Δ^ *P* < 0.05, ^ΔΔΔ^ *P* < 0.001, Compared with the Post-IBA group.

Compared with the SUM group, Na^+^, K^+^-ATPase activity in the Post-IBA and Post-H groups increased by 43.93% (*P* < 0.05) and 27.78% (*P* < 0.05), respectively; compared with the AUT/Pre-H and H group, Na^+^, K^+^-ATPase activity in the IB-A group decreased by 30.30% (*P* < 0.05) and 29.46% (*P* < 0.05), respectively; compared with the IB-A group, Na^+^, K^+^-ATPase activity in the Post-IBA and Post-H groups increased by 60.34% (*P* < 0.05) and 41.79% (*P* < 0.01), respectively (Fig. 1a).

In squirrels, the total Na^+^, K^+^-ATPase activity increased from summer to pre-hibernation, with a slight decline during hibernation and reaching its lowest level at arousal. The enzyme activity returned to high levels following arousal-sleep, and then decreased slightly during post-hibernation. Between the various groups, Na^+^, K^+^-ATPase activity was lowest in the IB-A group and highest in the Post-IBA group. Significant variation was found in total Ca^2+^-ATPase activity (*P* < 0.05) in the SOL muscle of squirrels during different periods of hibernation. Ca^2+^-ATPase activity in the IB-A group was lowest, with significant differences found in the other groups (*P* < 0.05); Ca^2+^-ATPase activity in the Post-IBA group was highest, and compared with the IB-A group, activity in the SUM, AUT/Pre-H, H, Post-IBA, and Post-H groups increased by 70.02% (*P* < 0.001), 73.25% (*P* < 0.001), 64.20% (*P* < 0.001), 141.26% (*P* < 0.001), and 64.18% (*P* < 0.001), respectively. Compared with the Post-IBA group, activity in the SUM, AUT/Pre-H, H, and Post-H groups decreased by 29.53% (*P* < 0.001), 28.19% (*P* < 0.001), 31.94% (*P* < 0.001), and 31.95% (*P* < 0.001), respectively (Fig. 1a).

### Effects of hibernation on Na^+^, K^+^-ATPase and Ca^2+^-ATPase activity in the EDL muscle of Daurian ground squirrels

Significant differences were found in Na^+^, K^+^-ATPase activity in the EDL muscle of squirrels. The Post-H group exhibited the highest activity, and compared with that of the AUT/Pre-H, IB-A, and Post-IBA groups, the activity in the Post-H group increased by 49.92% (*P* < 0.01), 43.83% (*P* < 0.01), and 35.06% (*P* < 0.05), respectively. No significant differences (*P* > 0.05) were observed among the other groups (Fig. 1b).

Compared with the SUM group, Ca^2+^-ATPase activity in the EDL muscle of squirrels in the AUT/Pre-H, H, IB-A, and Post-IBA groups decreased by 30.85% (*P* < 0.001), 33.79% (*P* < 0.001), 16.61% (*P* < 0.01), and 32.88% (*P* < 0.001), respectively, and in the Post-H group increased by 15.96% (*P* < 0.01); Ca^2+^-ATPase activity was highest in the Post-H group, which was the same as observed for Na^+^, K^+^-ATPase activity, and increased by 67.71% (*P* < 0.001), 75.15% (*P* < 0.001), 39.06% (*P* < 0.001), and 72.77% (*P* < 0.001) compared with the AUT/Pre-H, H, IB-A, and Post-IBA groups, respectively. Compared with the IB-A group, Ca^2+^-ATPase activity in the Post-IBA group decreased by 19.51% (*P* < 0.05). No significant differences were observed among the other groups (Fig. 1b).

### Effects of hindlimb-unloading on Na^+^, K^+^-ATPase and Ca^2+^-ATPase activity in the SOL muscle of Daurian ground squirrels

Compared with the AUT/Pre-H group, total Na^+^, K^+^-ATPase and Ca^2+^-ATPase activity in the SOL muscle of the AUT-HLU group showed no significant differences (*P* > 0.05) after two weeks of hindlimb-unloading (Fig. 1c).

Results demonstrated that hindlimb-unloading disuse had different influences on the SOL muscle of squirrels and rats, with no significant changes found in SOL cytosolic Ca^2+^ regulation.

### Effects of hindlimb-unloading on Na^+^, K^+^-ATPase and Ca^2+^-ATPase activity in the EDL muscle of Daurian ground squirrels

Compared with the AUT/Pre-H group, total Na^+^, K^+^-ATPase and Ca^2+^-ATPase activity in the EDL muscle of the AUT-HLU group showed no significant differences (*P* > 0.05) after two weeks of hindlimb-unloading (Fig. 1d).

As observed in the EDL muscle of rats, our results demonstrated that hindlimb-unloading had no significant effect on the squirrel EDL muscle in regard to cytosolic Ca^2+^ regulation.

### Effects of hindlimb-unloading on Na^+^, K^+^-ATPase and Ca^2+^-ATPase activity in the SOL muscle of rats

**Figure 2:**
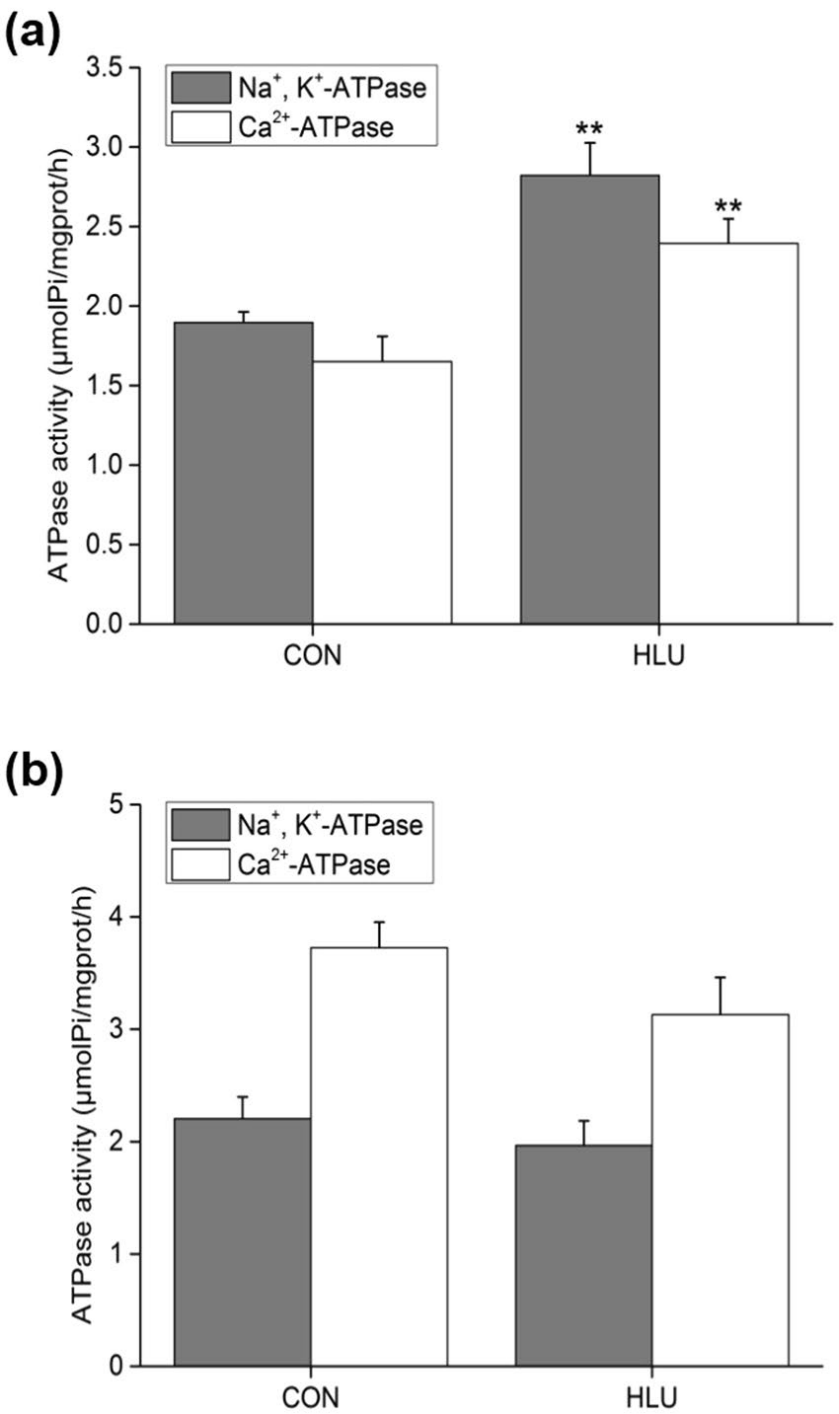
Effects of hindlimb-unloading on Na^+^, K^+^-ATPase and Ca^2+^-ATPase activity in the SOL and EDL muscles of rats (mean ± SD). (**a**) Activities in the SOL muscle. (**b**) Activities in the EDL muscle. **P < 0.01, Compared with the CON group.

Compared with the control (CON) group, Na^+^, K^+^-ATPase and Ca^2+^-ATPase activity in the SOL muscle of the HLU rat group exhibited highly significant differences after 14 d of hindlimb-unloading, increasing by 48.86% (*P* < 0.01) and 45.03% (*P* < 0.01), respectively (Fig. 2a).

Results showed that hindlimb-unloading increased total Na^+^, K^+^-ATPase and Ca^2+^-ATPase activity in the SOL muscle of rats.

### Effects of hindlimb-unloading on Na^+^, K^+^-ATPase and Ca^2+^-ATPase activity in the EDL muscle of rats

Compared with the CON group, Na^+^, K^+^-ATPase and Ca^2+^-ATPase activity in the EDL muscle of the HLU rat group exhibited no significant differences (*P* > 0.05) after two weeks of hindlimb-unloading (Fig. 2b).

Results also demonstrated that hindlimb-unloading of the non-anti-gravity EDL muscle of rats had no significant effect on cytosolic Ca^2+^ regulation.

### Effects of hibernation on SR Ca^2+^-ATPase activity in the SOL and EDL muscles of Daurian ground squirrels

**Figure 3:**
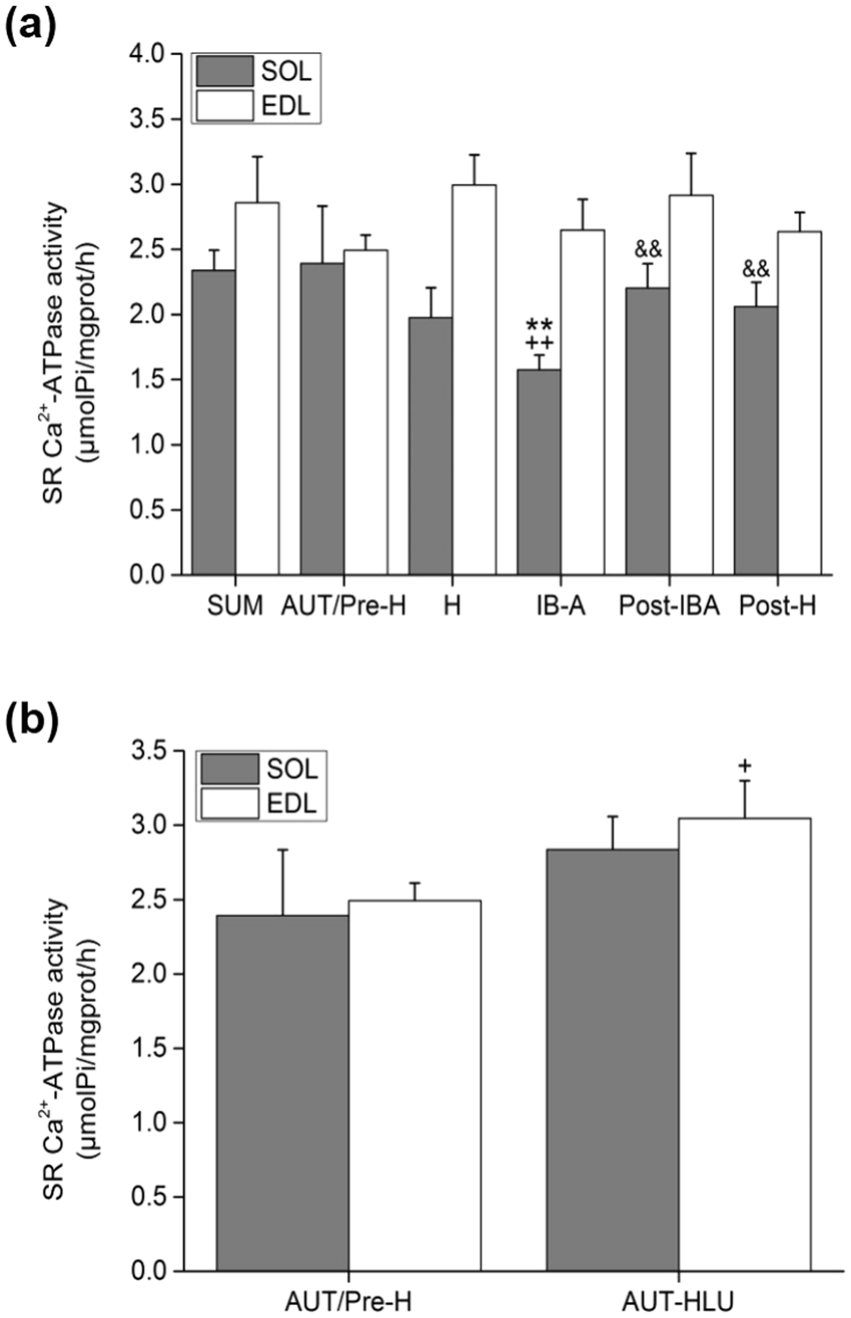
Effects of hibernation and hindlimb-unloading on SR Ca^2+^-ATPase activity in the SOL and EDL muscles of Daurian ground squirrels (mean ± SD). (**a**) Activity of hibernating ground squirrels. (**b**) Activity of hindlimb-unloading ground squirrels. **P < 0.01, Compared with the SUM group; ^+^ *P* < 0.05, ^++^ *P* < 0.01, Compared with the AUT/Pre-H group; ^&&^ *P* < 0.01, Compared with the IB-A group.

Ca^2+^-ATPase activity in the SOL-SR of the IB-A group was lower at different periods of hibernation. Compared with the SUM and Post-IBA groups, SR Ca^2+^-ATPase activity in the IB-A group decreased by 32.65% (*P* < 0.01) and 28.51% (*P* < 0.01), respectively, with significant differences found between the two groups. Compared with the AUT/Pre-H group, activity in the IB-A group decreased by 34.19% (*P* < 0.01). Compared with the IB-A group, activity in the Post-H group increased by 30.85% (*P* < 0.01). There were no significant differences between the AUT/Pre-H, H, and Post-H groups (*P* > 0.05) (Fig. 3a).

Results showed that Ca^2+^-ATPase activity in the SOL-ER of squirrels did not increase in hibernation, even during interbout arousal, and SR Ca^2+^-ATPase activity declined markedly, which differed from the hindlimb-unloading model and might be a mechanism involved in hibernating animal anti-disuse muscle atrophy.

Ca^2+^-ATPase activity in the EDL-SR of all squirrel groups showed no significant differences at different periods of hibernation (*P* > 0.05) (Fig. 3a). Results demonstrated that Ca^2+^-ATPase activity in the SR remained stable during different periods of hibernation.

### Effects of hindlimb-unloading on SR Ca^2+^-ATPase activity in the SOL and EDL muscles of Daurian ground squirrels

Compared with the AUT/Pre-H group, Ca^2+^-ATPase activity in the SOL-SR of squirrels in the AUT-HLU group demonstrated an increasing trend (*P* < 0.1), but no significant difference (*P* > 0.05). Compared with the AUT/Pre-H group, Ca^2+^-ATPase activity in the EDL-SR of the AUT-HLU group increased by 22.13% (*P* < 0.05) (Fig. 3b).

### Effects of hindlimb-unloading on SR Ca^2+^-ATPase activity in the SOL and EDL muscles of rats

**Figure 4:**
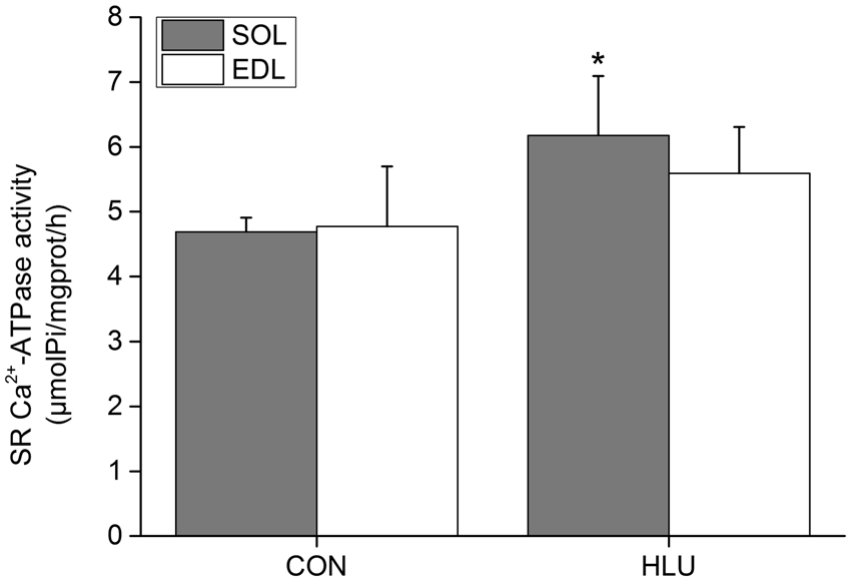
Effects of hindlimb-unloading on SR Ca^2+^-ATPase activity in the SOL and EDL muscles of rats (mean ± SD). *P < 0.05, Compared with the CON group.

Under a state of disuse, Ca^2+^-ATPase activity in the SOL-SR of the HLU rat group increased significantly by 31.76% (*P* < 0.05) compared with that of the CON group. However, there was no significant difference (*P* > 0.05) between the two groups in regard to Ca^2+^-ATPase activity in the EDL-SR (Fig. 4).

Furthermore, to resist cytosolic Ca^2+^ overload, Ca^2+^-ATPase activity in the SOL-SR of rats increased under disuse; however, hindlimb-unloading had no effect on Ca^2+^-ATPase activity in the non-anti-gravity EDL-SR.

### Effects of hibernation on SERCA2 protein expression in the SOL and EDL muscles of Daurian ground squirrels

**Figure 5:**
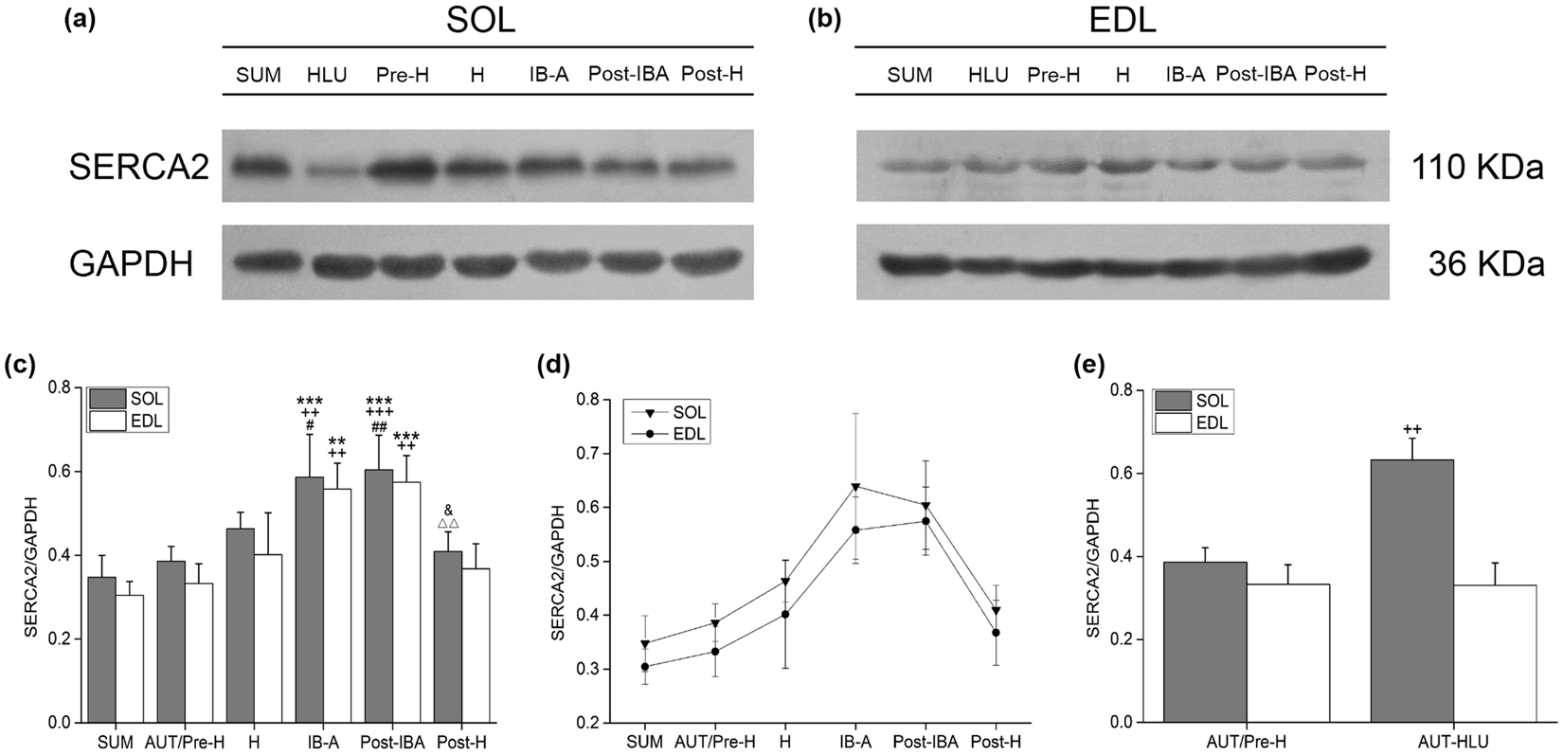
Changes in the protein levels of SERCA2 in the SOL and EDL muscles of Daurian ground squirrels during six hibernation periods (mean ± SD). (**a**) Representative immunoblots of SERCA2 in the SOL muscle during six hibernation periods. (**b**) Representative immunoblots of SERCA2 in the EDL muscle during six hibernation periods. (**c**) Ratio of SERCA2 to GAPDH in hibernating ground squirrels. (**d**) SERCA2 protein expression in the SOL and EDL muscles of Daurian ground squirrels during hibernation. (**e**) Ratio of SERCA2 to GAPDH in hindlimb-unloaded ground squirrels. **P < 0.01, ***P < 0.001, Compared with the SUM group; ^++^ *P* < 0.01, ^+++^ *P* < 0.001, Compared with the AUT/Pre-H group; ^#^ *P* < 0.05, ^##^ *P* < 0.01, Compared with the H group; ^&^ *P* < 0.05, Compared with the IB-A group; ^ΔΔ^ *P* < 0.01, Compared with the Post-IBA group.

Compared with the SUM group, the SERCA2 expression levels in the SOL muscle of the IB-A and Post-IBA groups significantly increased by 68.63% (*P* < 0.001) and 73.80% (*P* < 0.001), respectively. Compared with the AUT/Pre-H group, SERCA2 expression in the SOL muscle of the IB-A and Post-IBA groups significantly increased by 51.90% (*P* < 0.01) and 56.55% (*P* < 0.001), respectively. Compared with the H group, expression in the IB-A and Post-IBA groups significantly increased by 26.48% (*P* < 0.05) and 30.36% (*P* < 0.01), respectively. Compared with the Post-H group, expression in the IB-A and Post-IBA groups significantly increased by 43.20% (*P* < 0.05) and 47.58% (*P* < 0.01), respectively. Compared with the AUT/Pre-H group, expression in the Post-H group showed no significant difference (*P* > 0.05), and the expression between the IB-A and Post-IBA groups exhibited no significant difference (*P* > 0.05) (Fig. 5a,c).

There were no significant differences (*P* > 0.05) in SERCA2 expression in the EDL muscle of squirrels in the SUM, H, and Post-H groups. Compared with other groups, SERCA2 expression in the H group increased (*P* < 0.1). Compared with the SUM group, the expressions in the IB-A and Post-IBA groups were significantly different (*P* < 0.01), increasing by 83.34% (*P* < 0.01) and 88.76% (*P* < 0.001), respectively; however, there were no significant differences between the two groups (*P* > 0.05). Compared with the AUT/Pre-H group, the SERCA2 expression levels significantly increased by 67.80% (*P* < 0.01) and 72.77% (*P* < 0.01) in the IB-A and Post-IBA groups, respectively (Fig. 5b,c).

The two muscle disuse models (hindlimb-unloading and hibernation) had different effects on the SOL and EDL muscles of squirrels. SOL SERCA2 expression levels increased under both states, whereas EDL SERCA2 expression only increased during hibernation and produced no significant changes under hindlimb-unloading.

As seen in Fig. 5d, SERCA2 expression in the SOL and EDL muscles of squirrels during the entire hibernation showed a consistent trend. The expression of SERCA2 increased with hibernation, until post interbout arousal when it increased and showed the highest level. After the squirrels re-entered hibernation, the expression again decreased.

### Effects of hindlimb-unloading on SERCA2 protein expression in the SOL and EDL muscles of Daurian ground squirrels

Compared with the AUT/Pre-H group, SERCA2 expression in the SOL-SR of squirrels in the AUT-HLU group increased by 63.94% (*P* < 0.01) (Fig. 5 a,e); however, SERCA2 expression in the EDL-SR exhibited no significant changes (Fig. 5 b,e).

Furthermore, squirrel hindlimb-unloading (14 d) was found to affect the expression of SERCA2 in the SOL and EDL muscles, though more seriously in the SOL.

### Effects of hindlimb-unloading on SERCA2 protein expression in the SOL and EDL muscles of rats

**Figure 6:**
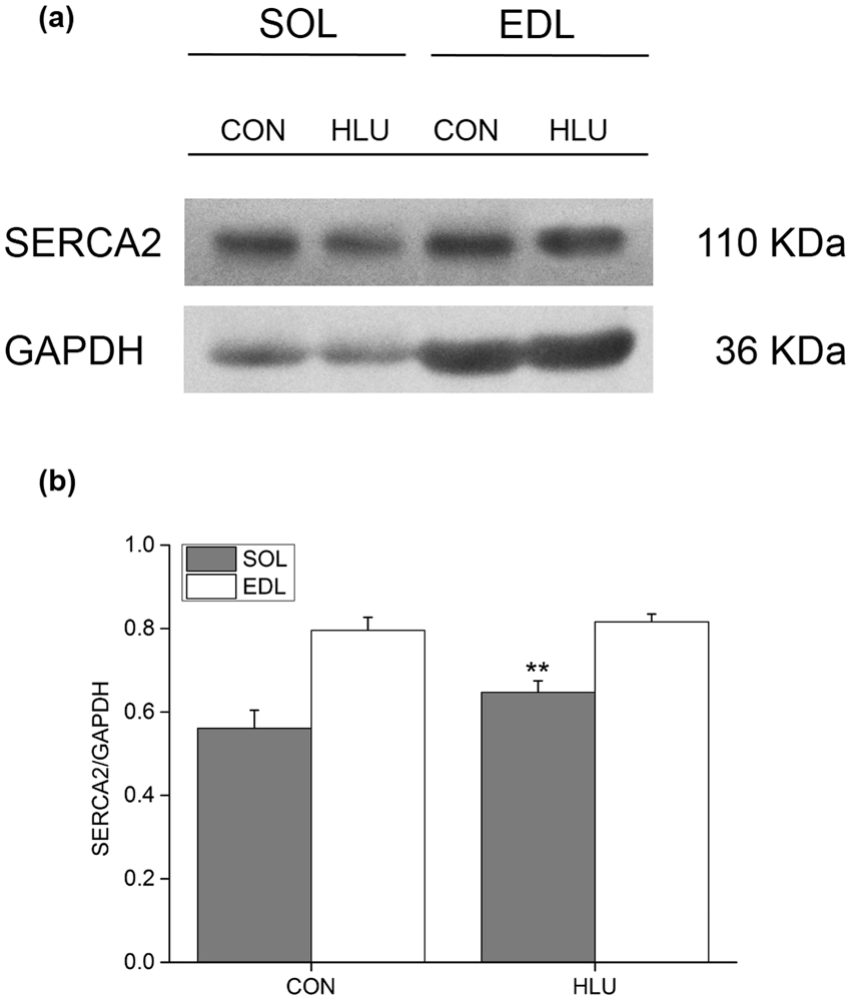
Changes in hindlimb-unloading on SERCA2 protein expression in the SOL and EDL muscles of rats (mean ± SD). (**a**) Representative immunoblots of SERCA2 in the SOL and EDL muscles of hindlimb-unloaded rats. (**b**) Ratio of SERCA2 to GAPDH in hindlimb-unloaded rats. **P < 0.01, Compared with the CON group.

Under a state of disuse, and compared with the CON group, the relative expression levels of SERCA2 in the SOL-SR of rats in the HLU group significantly increased by 15.28% (*P* < 0.01), but showed no significant differences in the EDL-SR (*P* > 0.05) (Fig. 6a,b).

In addition, under disuse, Ca^2+^-ATPase activity in the SOL-ER of rats increased mainly due to the increase in protein expression. Rear suspension had no effect on SERCA2 expression in the anti-gravity muscle toe long extensor.

### Effects of hibernation on p-PKA and PKA protein expression in the SOL and EDL muscles of Daurian ground squirrels

**Figure 7:**
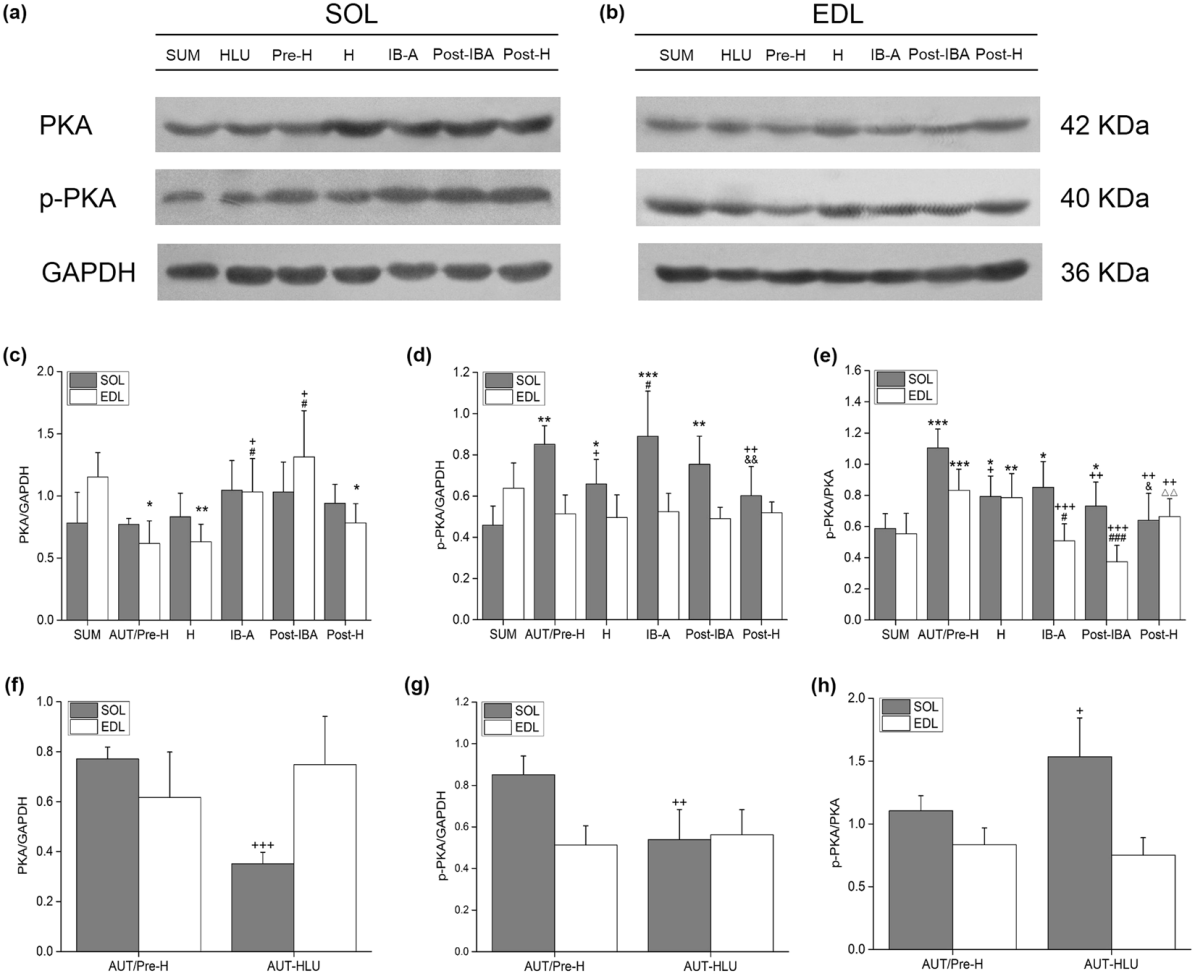
Changes in the PKA and p-PKA protein levels in the SOL and EDL muscles of Daurian ground squirrels during six hibernation periods (mean ± SD). (**a**) Representative immunoblots of PKA, p-PKA, and GAPDH in the SOL muscle during six hibernation periods. (**b**) Representative immunoblots of PKA, p-PKA, and GAPDH in the EDL muscle during six hibernation periods. (**c**) Ratio of PKA to GAPDH in hibernating ground squirrels. (**d**) Ratio of p-PKA to GAPDH in hibernating ground squirrels. (**e**) Ratio of p-PKA to PKA in hibernating ground squirrels. (**f**) Ratio of PKA to GAPDH in hindlimb-unloaded ground squirrels. (**g**) Ratio of p-PKA to GAPDH in hindlimb-unloaded ground squirrels. (**h**) Ratio of p-PKA to PKA in hindlimb-unloaded ground squirrels. *P < 0.05, **P < 0.01, ***P < 0.001, Compared with the SUM group; ^+^ *P* < 0.05, ^++^ *P* < 0.01, ^+++^ *P* < 0.001, Compared with the AUT/Pre-H group; ^#^ *P* < 0.05, ^###^ *P* < 0.001, Compared with the H group; ^&^ *P* < 0.05, ^&&^ *P* < 0.01, Compared with the IB-A group; ^ΔΔ^ *P* < 0.01, Compared with the Post-IBA group.

Compared with the SUM group, the PKA expression levels in the EDL muscle of the AUT/Pre-H, H, and Post-H groups significantly decreased by 46.50% (*P* < 0.05), 45.33% (*P* < 0.01), and 32.09% (*P* < 0.05), respectively. Compared with the AUT/Pre-H group, PKA expression in the EDL muscle of the IB-A and Post-IBA groups significantly increased by 67.28% (*P* < 0.05) and 112.99% (*P* < 0.05), respectively. Compared with the H group, expression in the IB-A and Post-IBA groups significantly increased by 63.68% (*P* < 0.05) and 108.41% (*P* < 0.05), respectively. No significant changes were found in the SOL muscle (*P* > 0.05) (Fig. 7c).

Compared with the SUM group, the p-PKA expression levels in the SOL muscle of the AUT/Pre-H, H, IB-A, and Post-IBA groups significantly increased by 85.57% (*P* < 0.01), 43.80% (*P* < 0.05), 93.96% (*P* < 0.001), and 64.45% (*P* < 0.01), respectively. Compared with the AUT/Pre-H group, p-PKA expression in the SOL muscle of the H and Post-H groups significantly decreased by 22.51% (*P* < 0.05) and 29.27% (*P* < 0.01), respectively. Compared with the IB-A group, expression in the H and Post-H groups significantly decreased by 25.86% (*P* < 0.05) and 32.33% (*P* < 0.01), respectively. No significant changes were found in the EDL (*P* > 0.05) (Fig. 7d).

Compared with the SUM group, the p-PKA/PKA ratio in the SOL muscle of the AUT/Pre-H, H, IB-A, and Post-IBA groups significantly increased by 88.04% (*P* < 0.001), 35.01% (*P* < 0.05), 45.02% (*P* < 0.05), and 24.51% (*P* < 0.05), respectively. Compared with the AUT/Pre-H group, the p-PKA/PKA ratio in the SOL muscle of the H, Post-IBA, and Post-H groups significantly decreased by 28.20% (*P* < 0.05), 33.78% (*P* < 0.01), and 41.99% (*P* < 0.01), respectively. Compared with the IB-A group, the ratio in the Post-H group significantly decreased by 24.79% (*P* < 0.05) (Fig. 7e).

Compared with the SUM group, the p-PKA/PKA ratio in the EDL muscle of the AUT/Pre-H and H groups significantly increased by 50.46% (*P* < 0.001) and 41.96% (*P* < 0.01), respectively. Compared with the AUT/Pre-H group, the p-PKA/PKA ratio in the EDL muscle of the IB-A, Post-IBA, and Post-H groups significantly decreased by 39.02% (*P* < 0.001), 55.13% (*P* < 0.001), and 20.36% (*P* < 0.01), respectively. Compared with the H group, the ratio in the IB-A and Post-IBA groups significantly decreased by 35.37% (*P* < 0.05) and 52.44% (*P* < 0.001), respectively. Compared with the Post-IBA group, the ratio in the Post-H group significantly increased by 77.48% (*P* < 0.01) (Fig. 7e).

### Effects of hindlimb-unloading on p-PKA and PKA protein expression in the SOL and EDL muscles of Daurian ground squirrels

Compared with AUT/Pre-H, the relative expression levels of PKA and p-PKA in the SOL of squirrels in the AUT-HLU group significantly decreased by 54.39% (*P* < 0.001) and 36.61% (*P* < 0.01), respectively, and the p-PKA/PKA ratio significantly increased in the AUT-HLU group by 39.00% (*P* < 0.05). However, no significant differences were observed between the AUT/Pre-H and AUT-HLU groups in the EDL (*P* > 0.05) (Fig. 7f,g and h).

### Effects of hindlimb-unloading on p-PKA and PKA protein expression in the SOL and EDL muscles of rats

**Figure 8:**
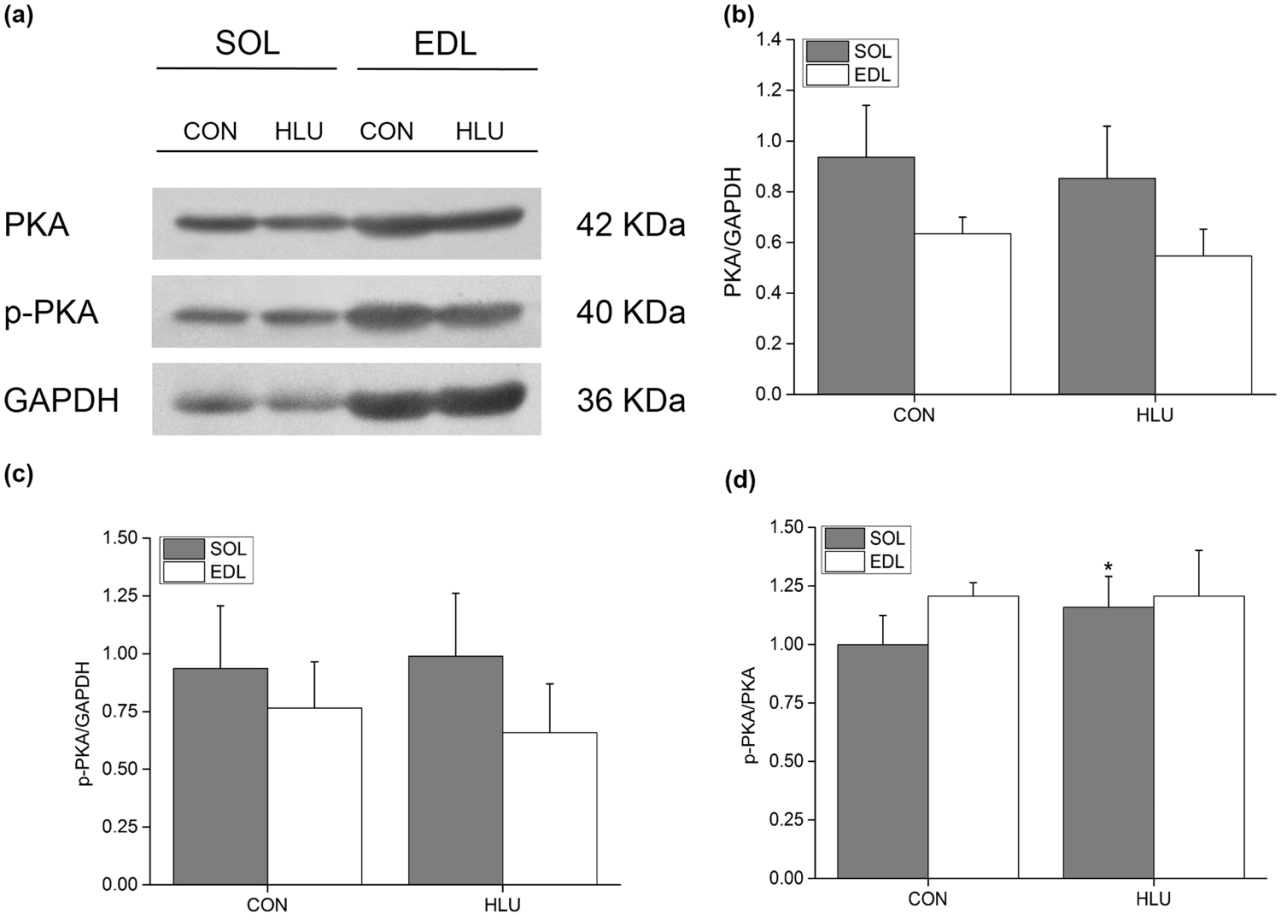
Changes in PKA and p-PKA protein expression in the SOL and EDL muscles of hindlimb-unloaded rats (mean ± SD). (**a**) Representative immunoblots of PKA, p-PKA, and GAPDH in the SOL and EDL muscles of hindlimb-unloaded rats. (**b**) Ratio of PKA to GAPDH in hindlimb-unloaded rats. (**c**) Ratio of p-PKA to GAPDH in hindlimb-unloaded rats. (**d**) Ratio of p-PKA to PKA in hindlimb-unloaded rats. *P < 0.05, Compared with the CON group.

Compared with the CON group, the relative expression levels of PKA and p-PKA in the SOL and EDL of rats in the HLU group showed no significant differences (*P* > 0.05) (Fig. 8b,c). However, the p-PKA/PKA ratio significantly increased by 15.98% (*P* < 0.05) in the SOL of the HLU group (Fig. 8d).

## Discussion

In the current study, we investigated the effects of hibernation inactivity and hindlimb-unloading on Na^+^, K^+^-ATPase, Ca^2+^-ATPase, SERCA2, PKA, and p-PKA in the skeletal muscle fibers of Daurian ground squirrels, and compared them with those in hindlimb-unloaded rats. Remarkable plasticity of Na^+^, K^+^-ATPase, Ca^2+^-ATPase, and SERCA were unexpectedly found. Moreover, up-regulation in the phosphorylation of PKA in the AUT/Pre-H and IB-A groups was also found, which might be a central mechanism of the plasticity of SERCA as its activity is widely dependent on the phosphorylation of phospholamban by p-PKA. Thus, these factors might contribute to disuse atrophy resistance in hibernators by decreasing the cytosolic Ca^2+^ concentration.

Intracellular Ca^2+^ homeostasis is an important part of the homeostasis system, and requires a dynamic balance between Ca^2+^ entrance into and exclusion from a cell, and between Ca^2+^ release from and uptake into organelles (especially the SR). There are two kinds of Ca^2+^-ATPase, one is plasma membrane Ca^2+^-ATPase (PMCA), which actively removes Ca^2+^ from cells, and the other is SERCA, which sequesters Ca^2+^ in the SR-Ca^2+^ store. Ca^2+^-ATPase plays an important role in avoiding excessive intracellular Ca^2+^, which can be detrimental to cell survival.

Na^+^, K^+^-ATPase actively removes Na^+^ from cells and establishes a Na^+^ concentration gradient inside and outside the cell. The Na^+^-Ca^2+^ exchanger (NCX), which transports three Na^+^ in for one Ca^2+^ out, is not directly coupled to ATP hydrolysis, but is instead driven across the cell membrane by total Na^+^ electrochemical gradients. Accordingly, Na^+^, K^+^-ATPase has a positive effect on Ca^2+^ removal in cells.

In this study, under prolonged hibernation inactivity, Na^+^, K^+^-ATPase activity was maintained in the slow SOL muscle of squirrels, but showed a significant 30.30% down-regulation in the IB-A group compared with the AUT/Pre-H group, and a 60.34% and 41.79% increase in the Post-IBA and Post-H groups, respectively, compared with the IB-A group. Similarly, Ca^2+^-ATPase activity was maintained in the slow SOL muscle of hibernating squirrels. Ca^2+^-ATPase activity in the IB-A group decreased by 39.10% compared with that in the H group, but increased in the Post-IBA group by 141.26% compared that in the IB-A group, and resumed pre-hibernation levels following final arousal from hibernation (Fig. 1a).

Both Na^+^, K^+^-ATPase and Ca^2+^-ATPase activity in the SOL muscle dropped to their lowest levels in the IB-A group, and reached their highest levels in the Post-IBA group. Moreover, Ca^2+^-ATPase activity showed a larger amplitude of vibration than that of Na^+^, K^+^-ATPase. Under interbout arousal, squirrels were active and had normal body temperature, so interbout exercise might be a factor in the underlying mechanism of intracellular Ca^2+^ homeostasis in hibernating animals by helping to resume normal Na^+^, K^+^-ATPase and Ca^2+^-ATPase activity. Considering that both Ca^2+^-ATPase and Na^+^ K^+^-ATPase have positive effects on inhibiting Ca^2+^ overload, the significant decrease in Na^+^, K^+^-ATP and Ca^2+^-ATP enzyme activity may have resulted in poorly maintained intracellular Ca^2+^ homeostasis, and forced interbout arousal. Therefore, Na^+^, K^+^-ATP and Ca^2+^-ATP enzyme activity levels or intracellular Ca^2+^ content could be considered as critical indexes of hibernator arousal from torpor.

The activities of Na^+^, K^+^-ATPase and Ca^2+^-ATPase were maintained in the fast EDL muscle in periods of hibernation, except for a significant increase in the Post-H group. Furthermore, the decreased Na^+^, K^+^-ATPase and Ca^2+^-ATPase activities observed in the SOL muscle of the IB-A group were not found in the EDL muscle (Fig. 1b), suggesting that Na^+^, K^+^-ATPase and Ca^2+^-ATPase activities were more sensitive to muscle disuse in slow SOL fibers than in fast EDL fibers during hibernation. However, the precise mechanism(s) underlying the differences between fast and slow muscle remains to be elucidated.

In the non-hibernating period, hindlimb-unloading showed no significant influence on the activity of the two enzymes in either the slow SOL or fast EDL muscles of squirrels (Fig. 1c,d). The fast EDL muscle results in squirrels were similar to those observed in hindlimb-unloaded rats, and confirmed that fast muscle was less affected by hindlimb-unloading (Figs 1 d and 2b). However, the slow SOL muscle results in squirrels were different from those found in hindlimb-unloaded rats, which showed a significant increase in both Na^+^, K^+^-ATPase and Ca^2+^-ATPase activity after 14 d of hindlimb-unloading (Figs 1 c and 2a). For non-hibernators, intracellular Ca^2+^ overload can be induced by different models of skeletal muscle disuse atrophy, and is involved in the mechanism of disuse atrophy^10, 24^. The elevated activity of the two enzymes in atrophied muscle might be activated by disuse-induced Ca^2+^ overload. This increased activity is consistent with previously reported shortened contraction and relaxation time^25^, and with the increased fast fiber ratio in disuse atrophied muscle^26^.

For Daurian ground squirrels, as typical hibernators, the activities of Na^+^, K^+^-ATPase and Ca^2+^-ATPase were maintained in the SOL muscle after 14 d of hindlimb-unloading, suggesting that cytosolic Ca^2+^ homeostasis of the muscle fiber was not easily affected by skeletal muscle disuse. Our previous research on Daurian ground squirrels showed that 14 d of hindlimb-unloading during a non-hibernating period did not lead to skeletal muscle atrophy^27^. Accordingly, the maintenance of Na^+^, K^+^-ATPase and Ca^2+^-ATPase activity might indicate stable Ca^2+^ homeostasis under muscle disuse conditions. Moreover, squirrels are likely pre-conditioned in this way to be resistant to disuse atrophy in non-hibernation periods.

Under long hibernation inactivity, the activity and protein expression of SR Ca^2+^-ATPase in the slow SOL muscle of squirrels was maintained in both the H and Post-H groups in the skeletal muscle fibers, suggesting striking homeostasis when compared with the pre-hibernation and summer active groups (Figs 3 a and 5c). However, SOL SR Ca^2+^-ATPase activity in the IB-A group showed a significant decrease of 34.19% (lowest level), followed by an increase of 39.88%, and recovery to pre-hibernation and summer active levels in the Post-IBA group, thus showing a similar change tendency with that of Na^+^, K^+^-ATPase and Ca^2+^-ATPase activity in the SOL fiber and suggesting that IB-A might play an important role in recovery of SR Ca^2+^-ATPase activity (Figs 1 a and 3a).

However, in contrast to the lowest level of SR Ca^2+^-ATPase activity in the IB-A group, the protein expression of SERCA type 2 (SERCA2) showed a significant increase (51.90% in the IB-A group, 56.55% in the Post-IBA group) in the SOL muscle when compared with the pre-hibernation group (Fig. 5c). These results suggest that up-regulation of the protein expression of SERCA2 in the IB-A group might be the basis for the rapid increase in SERCA2 activity in the Post-IBA group after less than 24 h of interbout arousal. Thus, the up-regulation of the SERCA2 protein might play an important role in avoiding Ca^2+^ overload by enhancing Ca^2+^ uptake into the SR, thereby contributing to the remarkable Ca^2+^ homeostasis in hibernation.

There were similar but smaller changes in the activity and protein expression of SERCA2 in the fast EDL muscle of squirrels. Somewhat different from the maintained Ca^2+^-ATPase activity in all periods of hibernation, the protein expression of SERCA2 showed a significant increase (67.80% in the IB-A group, 72.00% in the Post-IBA group), suggesting enhanced Ca^2+^ uptake into the SR and better Ca^2+^ homeostasis than that in the slow SOL muscle (Fig. 5c).

Consequently, locomotion during short interbout arousal possibly plays a role in resuming the pre-hibernation levels of activity and protein expression of SERCA2 under long hibernation inactivity. The meaningful change curve in activity of SR Ca^2+^-ATPase (Fig. 3a) suggests that when activity decreased to a minimum critical level or threshold, hibernating animals awoke from torpor and undertook interbout exercise, then re-entered torpor when the SR Ca^2+^-ATPase activity levels met their physiological demands. The increase in SERCA2 expression in the IB-A group might be the basis for the rapid increase in enzyme activity when the hibernators re-entered torpor (Post-IBA group). Accordingly, interbout exercise might be involved in the underlying mechanism of intracellular Ca^2+^ homeostasis in hibernating animals by the resumption of SR Ca^2+^-ATPase activity.

On the other hand, when we examined the effects of inactivity (hindlimb-unloading) during non-hibernation, the activity of SR Ca^2+^-ATPase in the fast EDL muscle showed a significant increase of 22.13% and SERCA2 expression in the slow SOL muscle showed a significant increase of 63.94% compared with that in the AUT/Pre-H group (Figs 3 b and 5e). These results differed from the increased activity and protein expression of SR Ca^2+^-ATPase only found in the slow SOL muscle of hindlimb-unloaded rats, suggesting a better adaptive ability to muscle disuse in hibernators (Figs 4 and 6b).

SERCA activity is widely dependent of the phosphorylation of by PKA, which accordingly plays an important role in the regulation of SERCA activity. In this study, the SERCA activity in the SOL muscle of the IB-A group was at its lowest level during the whole hibernation period (Fig. 3a), whereas the relative protein expressions of SERCA and p-PKA were at their higher levels (Fig. 5c and 7d). Therefore, we hypothesized that high-protein expression of p-PKA activated the high-protein expressed SERCA by phosphorylating, and was an important mechanism to restore SERCA activity to pre-hibernation levels in the Post-IBA. However, the protein expression of p-PKA in the EDL did not change significantly (Fig. 7d), which is consistent with the SERCA activity in the EDL (Fig. 3a). Thus, during interbout arousal, the hibernating animals improved SERCA activity by the up-regulation of p-PKA protein expression, thereby enhancing the backtracking of Ca^2+^ into the SR and reducing high Ca^2+^ concentration to restore homeostasis. It might be that hibernating animals maintain cytoplasmic Ca^2+^ homeostasis by maintaining remarkable SERCA plasticity.

The PKA and p-PKA protein expressions decreased significantly in the SOL of the AUT-HLU group after 14 d of hindlimb-unloading. These results suggest that although the SERCA activity showed no significant change, but the ratio of p-PKA/PKA showed a significant increasing, the squirrels had a high potential for up-regulating the activity of SERCA. On the other hand, the significantly reduced protein expression levels of PKA and p-PKA suggest that skeletal muscle disuse after 14 d of hindlimb-unloading might not cause significant cytoplasmic Ca^2+^ homeostasis imbalance, and thus did not show significant changes in the expression levels of the relevant regulatory factors.

Compared with the CON group, the protein expressions of PKA and p-PKA showed no significant changes in the SOL and EDL; however, the p-PKA/PKA ratio increased significantly in the SOL of the HLU group. This was consistent with the significant increase in SERCA activity observed in HLU rats, and suggests that PKA is an important factor in the regulation of SERCA activity in rat skeletal muscle. On the other hand, significant cytoplasmic Ca^2+^ homeostasis imbalance after 14 d of hindlimb-unloading was observed, and caused significant changes in the relevant regulatory factors.

These rat and ground squirrel results were in accordance with their skeletal muscle performance under disuse conditions; that is, significant atrophy in rats and limited atrophy in ground squirrels^27^. Furthermore, these findings suggest that skeletal muscle atrophy protection in hibernators might be pre-conditioned in non-hibernation periods by a better ability to maintain Ca^2+^ homeostasis.

In summary, under long hibernation inactivity, the activity and protein expression of SERCA2 showed good plasticity, apart from the decreased activity accompanying increased protein expression in the SOL muscle of the IB-A group, and the protein expression of p-PKA was also up-regulated in this stage in the SOL. Furthermore, subsequent increased activity and higher protein expression in the SOL muscle of the Post-IBA group strongly suggests the importance of interbout arousal for hibernators to maintain Ca^2+^ homeostasis, and that it is an important mechanism to maintain SERCA plasticity during interbout arousal. Under muscle disuse conditions induced by hindlimb-unloading, the activity and protein expression of SERCA2 in squirrels showed better homeostasis than that observed in the non-hibernating rats. Combined with the performance of skeletal muscle structure and function in the non-hibernation period, we concluded that the unusual regulation capacity of cytoplasmic Ca^2+^ might be involved in preventing muscle disuse atrophy in hibernators and might be partly preconditioned in non-hibernation periods.

## Conclusions

Unusual regulation capacity of cytoplasmic Ca^2+^ might be involved in preventing skeletal muscle disuse atrophy in hibernating Daurian ground squirrels. Locomotion during short interbout arousal could be an important factor in maintaining Ca^2+^ homeostasis by resuming pre-hibernation levels of Na^+^, K^+^-ATPase, Ca^2+^-ATPase and SERCA2 after long hibernation inactivity. Moreover, hibernators might be preconditioned against disuse atrophy during non-hibernation periods by superior Ca^2+^ homeostasis. Specifically, muscle disuse induced by hindlimb-unloading showed fewer negative effects on Na^+^, K^+^-ATPase, Ca^2+^-ATPase, and SERCA2 in the skeletal muscles of squirrels compared with that in the skeletal muscles of rats.

## Limitations of the study

As the adrenergic pathway regulates SERCA activity through the up-regulation of PKA, determining changes in this pathway is necessary for an in-depth exploration of the regulatory mechanism of SERCA activity. However, due to the limited amount of muscle samples from hibernating ground squirrels, which could only be collected once a year, the adrenergic pathway was not investigated in the present study, but will carried out on ground squirrels in our future research.

## Materials and Methods

### Animals and groups

We captured healthy adult Daurian ground squirrels of both sexes from the Weinan region in the Shaanxi province of China. All animal procedures, as well as care and handling, were approved by the Laboratory Animal Care Committee of the China Ministry of Health.

The animals were sorted by sex and placed into conventional plastic cages (0.35 × 0.3 × 0.45 m^3^), with two animals per cage. Animals were acclimated for one to two months after collection before being divided into the relevant study groups. The colony room was maintained at 18–20 °C, with lighting adjusted every day to mimic sunrise and sunset. Cages were lined with wood chips to provide a more natural base. Squirrels were provided with rodent food blocks and fresh fruit and vegetables daily, with water supplied *ad libitum*. When the hibernation period commenced, three groups were placed in a hibernaculum cold room (4–6 °C and 2 L:22D dark). Each animal was deemed to have entered torpor when their body temperature fell below 9 °C and general body movement (judged by placing sawdust on their backs) ceased, at which time the torpor date was recorded. Thermal imaging was performed using a visual thermometer (Fluke VT04 Visual IR Thermometer, USA) to determine body temperature. Based on prior yearly records, our previously studied hibernators were found to return to hibernation after one to two days of interbout arousal. In the present study, animals awake for more than two days following a hibernation bout were assigned to the Post-H group. Observations were conducted every day during the entire experimental period.

After matching for body mass, the animals were randomly assigned to one of seven groups (n = 10/group): summer group (SUM), autumn/pre-hibernation group (AUT/Pre-H), autumn hindlimb-unloaded group (AUT-HLU), hibernation group (H), interbout arousal group (IB-A), torpor after interbout arousal group (Post-IBA), and post-hibernation group (Post-H).

Adult female Sprague-Dawley rats (200–220 g) were obtained from the Experimental Animal Center of the Medical College of Xi’an Jiaotong University, Xi’an, China. After matching for body mass, the animals were randomly assigned to one of two groups (N = 8/group), that is, the synchronous control (CON) or the hindlimb-unloaded (HLU) group. This study was approved by the Ethical Committee of Northwest University and studies were performed in accordance with the Guide for the Care and Use of Laboratory Animals defined by the Ethical Committee of Northwest University. All procedures related to animals were approved by the Laboratory Animal Care Committee of the P. R. China Ministry of Health.

Animal welfare and experimental procedures were carried out in accordance with the Guide for the Care and Use of Laboratory Animals (Ministry of Science and Technology of China, 2006). All procedures were approved by the Laboratory Animal Care Committee of the P. R. China Ministry of Health and the Institutional Animal Ethics Committee of Xi’an Northwest University.

### Hindlimb-unloaded animal models

Hindlimb-unloading lasted 14 days. Orthopedic tape-adhesive plaster, covering less than half a clean and dry tail, was attached by a shoelace to the top of the cage where a swivel joint allowed 360° rotation. The lace length was adjusted to ensure body inclination of 30° from the horizontal plane, but which still allowed free forelimb movement. The suspension cages were 0.35 × 0.3 × 0.45 m^3^ in size.

Rats were raised in conventional plastic cages (0.35 × 0.3 × 0.45 m^3^) separately, and were fed standard laboratory rat chow and provided with water *ad libitum*. Their housing environment was maintained at 20–25 °C with a 12/12-h light-dark cycle. The welfare and experimental procedures related to rats were carried out in accordance with the Guide for the Care and Use of Laboratory Animals (Ministry of Science and Technology of China, 2006). All procedures were approved by the Laboratory Animal Care Committee of the P. R. China Ministry of Health and the Institutional Animal Ethics Committee of Xi’an Northwest University.

### Muscle collection

Animals were anaesthetized by an intraperitoneal injection of 90 mg/kg of sodium pentobarbital for squirrels and 45 mg/kg of sodium pentobarbital for rats. Hindlimb SOL and EDL muscles were excised from both legs; body weight and wet mass of the SOL and EDL muscles were recorded, and the activities of Na^+^, K^+^-ATPase and Ca^2+^-ATPase in these skeletal muscles were determined by the Fiske-Subbarow method. The remaining samples were stored at −80 °C until further processing. Following surgical intervention, the animals were sacrificed by sodium pentobarbital overdose injection. Animal experimental procedures were carried out in accordance with the Guide for the Care and Use of Laboratory Animals (Ministry of Science and Technology of China, 2006). All procedures were approved by the Laboratory Animal Care Committee of the P. R. China Ministry of Health and the Institutional Animal Ethics Committee of Xi’an Northwest University.

### Muscle Na^+^, K^+^-ATPase and Ca^2+^-ATPase activity assay

The SOL and EDL muscles were removed and cleaned of fat and connective tissue. Samples (0.04 g) were carefully excised from the muscle mid-belly, placed in 8% (wt/vol) ice-cold buffer, and homogenized [1 g muscle/4 volumes 10 mM homogenate buffer (250 mM sucrose, 10 mM NaN3, 5 mM HEPES, 0.2 mM PMSF, pH 7.50)] for 1 min at 4 °C in a Polytron homogenizer (PT2100, Switzerland). The 10% crude homogenates were then centrifuged at 1,600 g for 10 min (2 °C), and the supernatant was removed and stored at −80 °C until further processing. Total protein concentration was determined by the Bradford method. The activities of Na^+^, K^+^-ATPase and Ca^2+^-ATPase were determined using a spectrophotometer with commercial colorimetric assay kits (Jiancheng Bioengineering Institute, Nanjing, China), as per the manufacturer’s instructions. One unit of Na^+^, K^+^-ATPase or Ca^2+^-ATPase activity was defined as 1 µM Pi released after 1 h in 1 mg of protein at 37 °C.

### Western blot analysis

We extracted total protein from the SOL and EDL muscles of animals via homogenization in SDS-PAGE sample buffer (1% SDS). The muscle protein extracts were resolved by SDS-PAGE using Laemmli gels (10% gel with a 37.5:1 acrylamide to bisacrylamide ratio and 12% gel with a 29:1 acrylamide to bisacrylamide ratio for SERCA2, PKA, p-PKA, and GAPDH, respectively). Following electrophoresis, the resolved proteins were transferred electrically to nitrocellulose membranes (Roche, China) with a pore size of 0.45 um using a Bio-Rad semi-dry transfer apparatus (Bio-Rad, USA). The nitrocellulose membranes were then blocked with 1% bovine serum albumin (BSA) in Tris-buffered saline (TBS; 150 mM NaCl, 50 mM Tris-HCl, pH 7.5) and incubated with rabbit polyclonal anti-SERCA2 ATPase antibody (1: 20000; Abcam, UK), anti-cAMP Protein Kinase Catalytic subunit antibody (1:50000; Abcam, UK), anti-PKA alpha/beta/gamma (catalytic subunit) (phosphor T197) antibody (1:3750; Abcam, UK), and rabbit monoclonal anti-GAPDH (1: 5000; Bioworld Technology, USA) in TBS containing 0.1% BSA at 4 °C overnight. The nitrocellulose membranes were then incubated for 90 min at room temperature with IRDye 680 CW goat-anti rabbit or IRDye 800 CW goat-anti rabbit secondary anti-bodies (1:10,000), and finally visualized using an Odyssey scanner (LI-COR Biosciences, Lincoln, NE, USA). Blot quantification analysis was performed using NIH Image J software.

### Statistical analysis

Statistically significant differences (SPSS 21.0) were determined using one-way analysis of variance (ANOVA). Differences between groups were established using Fisher’s least-significant difference (LSD) post hoc test. When homogeneity was not detected, the ANOVA-Dunnett’s T3 method was used. Body weight loss was assessed using paired-samples *t*-test. Results were considered statistically significant at *P* < 0.05, with a tendency assumed at 0.05 < *P* < 0.1.
